# Vascular Endothelial Cell Biology: An Update

**DOI:** 10.3390/ijms20184411

**Published:** 2019-09-07

**Authors:** Anne Krüger-Genge, Anna Blocki, Ralf-Peter Franke, Friedrich Jung

**Affiliations:** 1 Department of Biomaterials and Healthcare, Division of Life Science and Bioprocesses, Fraunhofer Institute for Applied Polymer Research (IAP), 14476 Potsdam-Golm, Germany; 2 Department of Anesthesia, Pain Management and Perioperative Medicine, Faculty of Medicine, Dalhousie University, Halifax, NS B3H 2Y9, Canada; 3 Institute for Tissue Engineering and Regenerative Medicine (ITERM), School of Biomedical Sciences (SBS), Chinese University of Hong Kong (CUHK), New Territories, Hong Kong, China; 4 Central Institute for Biomedical Technology, Dep. Biomaterials, University of Ulm, Albert-Einstein-Allee 47, 89081 Ulm, Germany; 5 Institute of Biotechnology, Molecular Cell Biology, Brandenburg University of Technology, 01968 Senftenberg, Germany

**Keywords:** endothelium, shear stress, angiogenesis, glycocalyx, thrombosis

## Abstract

The vascular endothelium, a monolayer of endothelial cells (EC), constitutes the inner cellular lining of arteries, veins and capillaries and therefore is in direct contact with the components and cells of blood. The endothelium is not only a mere barrier between blood and tissues but also an endocrine organ. It actively controls the degree of vascular relaxation and constriction, and the extravasation of solutes, fluid, macromolecules and hormones, as well as that of platelets and blood cells. Through control of vascular tone, EC regulate the regional blood flow. They also direct inflammatory cells to foreign materials, areas in need of repair or defense against infections. In addition, EC are important in controlling blood fluidity, platelet adhesion and aggregation, leukocyte activation, adhesion, and transmigration. They also tightly keep the balance between coagulation and fibrinolysis and play a major role in the regulation of immune responses, inflammation and angiogenesis. To fulfill these different tasks, EC are heterogeneous and perform distinctly in the various organs and along the vascular tree. Important morphological, physiological and phenotypic differences between EC in the different parts of the arterial tree as well as between arteries and veins optimally support their specified functions in these vascular areas. This review updates the current knowledge about the morphology and function of endothelial cells, particularly their differences in different localizations around the body paying attention specifically to their different responses to physical, biochemical and environmental stimuli considering the different origins of the EC.

## 1. Introduction 

The vascular endothelium is the inner-most structure that coats the interior walls of arteries, capillaries and veins. Endothelial cells (EC) were described to anchor to an 80-nm-thick basal lamina (BL). Both EC and BL constitute the vascular intima, establishing a hemocompatible surface, estimated a total combined surface area of 3000–6000 m^2^ in the human body, comprising 1 to 6 × 10^13^ EC [1,2]. From their first description in 1865 until the early 1970s, this monolayer was regarded as a mere inert barrier separating blood cells from the surrounding tissue. 

**Figure 1:**
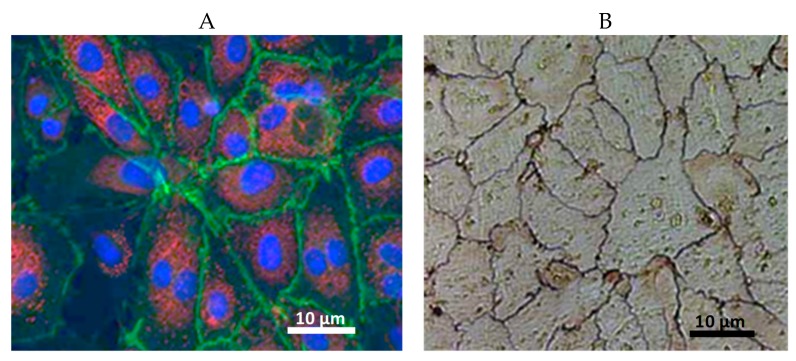
(**A**) Immunostaining of an endothelial cell monolayer (cell nuclei in blue, von Willebrand factor in red, vinculin in green); (**B**) Endothelial cell borders from the confluent endothelial cell monolayer are stained according to Ranvier with AgNO_3_ (400-fold primary magnification).

ECs are polarized cells: their luminal membrane is directly exposed to blood constituents and circulating cells, while the basolateral surface is separated from surrounding tissues by a glycoprotein basement membrane which is secreted and anchored to their cell membrane by EC themselves. The shape of the EC varies along the vascular tree, but they are generally thin and slightly elongated, their dimensions described to be roughly 30–50 µm in length, 10–30 µm wide and a thickness of 0.1–10 µm. EC are orientated along the axis of the vessel in the blood vessel wall in order to minimize the shear stress exerted by the flowing blood. In vitro EC monolayers show a characteristic cobble-stone pattern. Figure 1A shows a monolayer of human venous EC stained threefold (cell nuclei stained in blue, von Willebrand factor in red, and vinculin stained in green). In Figure 1B, silver nitrate staining shows the EC borders marked by typical zigzag lines due to interdigitating at the EC monolayer [3]. 

Vinculin is a membrane-cytoskeletal protein in focal adhesion plaques that is involved in cell-cell and cell-matrix junctions by linking integrin adhesion molecules to the actin cytoskeleton [4].

Considering the heterogeneity of the vascular system, it is hard to understand that EC, which are the major players of vascular performance, could be regarded as an inert cell layer. Big diameter vessels (arteries, veins, arterioles and venules) conduct the blood from the heart to organs and tissues and back, practically without a greater loss of blood fluid or cells across the EC layer under physiological conditions. However, a great variability in the permeability of these vessels is described. In spite of great differences and variabilities in blood pressures (arterial versus venous, low work load with low heart pressure/pulse rate versus high work load with high heart pressure/pulse rate), this low permeability is maintained by EC.

Capillaries, on the contrary, are vessels meant for the exchange of fluids, solutes and condensed matter between the intra- and extra-vascular compartments [5]. The permeability of vessels of the blood-brain-barrier is extremely low, of arteries and veins very low and of arterioles and venules very low to low [5,6,7]. Great variability in permeability is also found in different capillary regions. In most capillaries, the pressure values range between 0 to 25 mmHg. However, in extreme cases, such as the capillaries of kidneys’ glomeruli, pressure can amount to 50 mmHg. There is also a great variability in the transport capabilities across endothelial layers in exchange vessels. While most capillaries allow export and import of blood plasma under normal physiological conditions, there is an enhanced exchange of fluids, solutes and condensed matter in the capillaries of kidney glomeruli, through the specialized nano-filtration process. Sinusoidal vessels of the spleen allow for the transfer of blood cells and elimination of senescent red blood cells. In analogy to nano-filtration in kidney glomerular capillaries, the process in the spleen could be regarded as micro-filtration.

Moreover, some regions of the capillary system (e.g., lungs, gut, liver) are intermediate depots blood as a whole or specific blood cells such as erythrocytes, leukocytes and platelets. There are different mechanisms which allow for the temporary docking, storage and release of different cells in the respective capillary regions.

One further essential “on-demand” mechanism is the endothelial cell-regulated recruitment of capillaries. This increased number of perfused capillaries is induced to response to a stimulus such as an increase in muscular work [8]. 

Variability is also found in the general vascular structure, comprising of the hierarchical organization of heart, arteries, arterioles, capillaries, followed by venules, veins and heart, again. This succession of vessel types varies in specialized vascular networks. In the kidney glomeruli, it is organized in the structure of arterioles, capillaries, arterioles, then again by capillaries, venules and veins. 

These are few of the many examples of the complex of the vascular system that mirror the variety of situations and processes that ECs face under physiological conditions. Under pathophysiological conditions, this complexity becomes exaggerated. 

Evidently, EC have many functions that are specific to their location and they exhibit considerable phenotypic heterogeneity across the vascular bed [9,10]. The endothelium actively controls the degree of vascular relaxation/constriction, the extravasation of solutes, fluid, hormones, and macromolecules [11], as well as that of platelets and blood cells [12,13]. In addition, EC are actively involved in the suppression of the intermediate vascular layer cells (tunica media; i.e., vascular smooth muscle cells) to avoid the outgrowing into the tunica intima layer disturbing normal vascular function.

The discovery of prostacyclin [14], its synthesis in EC [15] and the groundbreaking report from Furchgott & Zawadski about its active role of the endothelium on the vasodilation of the vasculature [16] have pointed to the endothelium as a key player in homeostasis of many pathophysiological processes. Recognized now, the endothelium is described as a dynamic organ regulating its environment and responding to external stresses thereby playing not merely a passive role. It also became clear that the endothelium is an active metabolic and endocrine organ [17]. Especially in consideration with the fact that EC are responsible for the supply of tissues with oxygen by synthesis and release of relaxing and contracting factors modulating blood flow rate. These factors comprise (1) endothelial-derived hyperpolarizing factors like nitric oxide (NO) and EDHF, (2) metabolites of arachidonic acid that signal via cyclooxygenases, lipoxygenases and cytochrome P450 pathways, and (3) peptides like endothelin, urotensin, C-type natriuretic peptide (CNP), adrenomedullin, adenosine, purines, reactive oxygen species and others. An imbalance in the synthesis and/or release of such mediators results in endothelial dysfunction [18,19,20] and have also been reported to be important in cardiovascular pathologies such as hypertension, diabetes mellitus and atherosclerosis.

## 2. Luminal Endothelial Cell Surface, the Glycocalyx

The apical or luminal plasma membrane of EC carries the site for the glycocalyx (GCX), which is a complex network of macromolecules [21]. The glycocalyx has a multilayer structure which reduces EC contact with cellular and macromolecular blood components. The bulk of the glycocalyx is formed by glycoproteins and proteoglycans (PG) [22]. Its composition and dimensions fluctuate with changing shear forces from turbulent blood, leading to loss and re-synthesis of GCX constituents [23]. The synthesis of the glycocalyx is a complex process, involving multiple enzymatic pathways [24]. Several factors regulating its shedding including local pH and mechanical stimuli [25]. Hyaluronan synthase, for example, is capable to produce one hyaluronan molecule of 240 kDa roughly within 5 min [26]. Potter et al. described that the rate of synthesis decreased with a decrease of hyaluronan synthase expression as well as with an increase in molecular weight of hyaluronan [27]. The authors concluded that an EC should be able to produce enough hyaluronan to constitute a hemodynamically relevant layer within 24 h [28]. Also, the removal of hyaluronan from the circulation is very efficient, with a half-life of 2–6 min and a total turnover of 10–100 mg/day in the adult human [29]. Strikingly, in patients undergoing cardiac surgery with cardio-pulmonary bypass (CPB), the use of a pulsatile flow was described to be essential for the recovery of the GCX after injury upon onset of extracorporeal circulation within four hours. There was no recovery with non-pulsatile flow during CPB [30].

The glycocalyx forms a luminal mesh that provides EC with a framework to bind plasma proteins and soluble glycosaminoglycans (GAG). The GCX was shown to become physiologically active once plasma constituents bind to or immerse into the glycocalyx [25]. Negatively charged GAG side chains are attached to the PG’s core protein. This PG differ in the size of their core proteins and the number of GAG side chains including their binding to the cell membrane. The most frequent GAG (50–90%) are heparan-sulfates (HS) [25]. Other GAG found are hyaluronic acid and chondroitin-, dermatan-, and keratan-sulfates. Heparan sulfates are found on several core proteins including perlecans, glypicans, and syndecans. Perlecan is a large heparan sulfate proteoglycan, which is found in the basement membrane. Glypicans belong to the cell surface HS proteoglycans, which have a glycosylphosphatidylinositol anchor [31]. The syndecan family belongs to the transmembrane proteoglycans found in the GCX that are shed in a soluble form when the GCX becomes disordered. Each syndecan consists of the following components: an extracellular domain containing GAG attachment sites, a single pass transmembrane domain, and a short cytoplasmic domain including phosphorylation sites. Further core proteins, such as decorins, biglycans, versicans, and mimecans, are dermatan sulfate-bearing or chondroitin sulfate-bearing proteoglycans [32]. Differing from the other GAG, hyaluronic acid is the only non-sulfated and protein-core free GAG.

The GCX participates in different vascular functions. It plays a role in forming a barrier [33] though it is charged and complexed mesh structure acting as a macromolecular sieve [34]. The negative charge repels negatively charged molecules, as well as white and red blood cells and platelets away from the GCX. Macromolecules larger than 70 kDa are excluded from the tight mesh structure in the GCX, while Albumin (67 kDa) despite its overall negative charge at neutral pH still binds tightly to the GCX [35], due to its amphoteric character. By this binding, the hydraulic conductivity across the vascular barrier are reduced [36]. 

The glycocalyx, through its intracellular protein domain, can also act as a mechano-transducer, enabling EC to sense mechanical stress [22,33]. Conformational changes in the glycocalyx as well as shedding of microparticles [37] (e.g., induced by blood flow) contribute to the regulation of vasomotor tone and thereby the distribution of oxygen by triggering the release of nitric oxide. By this rheological mechanism, the glycocalyx contributes to the maintenance of homeostasis in the peripheral tissues [38].

## 3. Vascular Endothelium and Shear Stress

Due to their strategic location, the vascular EC are able to sense hemodynamic changes and blood-borne signals and to respond by the release of vasoactive substances. Under physiological conditions, endothelium-derived relaxing and contracting factors are balanced, so that vascular homeostasis is maintained marginally in favour of vasodilation [39]. In addition to changes in cell morphology, EC respond to defined flow stimuli instantaneously with electrochemical activities and gene expression [40,41]. The most significant of these changes is that NO release increases when shear stress increases. This occurs by rapid eNOS activation with upregulation of eNOS gene expression and the transcription activation of the eNOS promoter [42].

**Figure 2:**
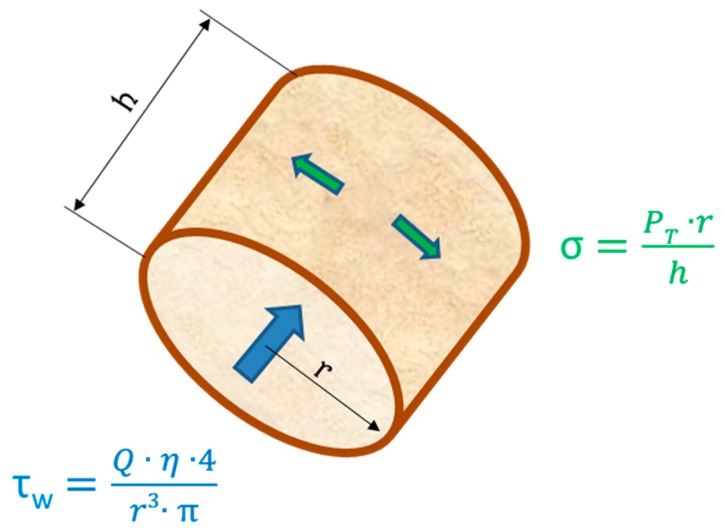
Hemodynamic stresses (τ_W_: wall shear stress, σ: circumferential wall stress) acting on the endothelial cell monolayer. (Q: blood volume flow, η: blood viscosity, r vessel radius, P_T_: transmural pressure difference).

The endothelium is exposed to different mechanical and hemodynamical forces: (i) radial forces caused by the intravascular pressure, (ii) tangential forces (P_T_) in the vessel wall caused by the balance between cell-cell contacts and vasomotion of the vessel, and (iii) axial shear forces caused by the friction of the flowing blood against the vessel wall (see Figure 2). 

EC, typically 1 to 100 dynes/cm^2^ which exposed to arterial levels of steady or pulsatile unidirectional shear stress in vitro [43] adopt an anti-inflammatory, anti-thrombotic and anti-proliferative phenotype. They are different from cells in static culture [44]. These form a functionally-confluent cell monolayer [45]. Cell-cell junctions [46], heterotrimeric G-proteins [47], primary cilia [48], caveolae [49], integrins [50] and the GCX [51] enable EC to detect and react to the shear forces of the flowing blood. EC grown upon ECM under static conditions in vitro and exposed to shear stress lengthened and aligned their long axis in flow direction and developed stress fibers in central parts of the cells [52]. However, this only appeared at arterial levels of shear stress, while venous levels of shear stress had no significant effect on the stress fiber induction [45,52].

Previous research has identified a mechano-sensory complex at cell-cell junctions, consisting of PECAM-1, VE-cadherin, and VEGFR-2, which is required for the activation of a number of shear-dependent signalling pathways, mediating both cell alignment and activation of many pro-atherosclerotic pathways [46]. In this complex, PECAM-1 is the primary mechano-transducer stimulated by external forces to initiate signalling. VE-cadherin is thought to act as an adapter, moving VEGFR-2 next to PECAM-1, thereby facilitating VEGFR-2 transactivation by a Tyrosine family kinase. Activated VEGFR-2 recruits PI3-kinase and mediates the activation of a protein kinase B (Akt) and eNOS [46,53]. 

The transcription factor KLF2 (Kruppel like factor 2) [54], which suppress an inflammatory activation, a feature missing in atherosclerosis-prone areas and characterized by a disturbed blood flow [55,56,57,58], is induced by arterial shear forces.

Shear forces also have an impact on the nature of thrombi, which are white and platelet-rich in the arterial tree but are red and carry more fibrin in veins. Venous vessels often contain valves, especially in the lower limbs. Due to the disturbed flow, local hypoxia or other unknown reasons, here often venous thrombi are initiated. The high presence of tissue-type plasminogen activator (t-PA), a regulator of fibrinolysis may contribute to the aspect that these thrombi have only little contact with the endothelium, while extending and growing in size until they form large emboli in the bloodstream.

These observations have led to the concept that high shear, typical for straight segments of arteries, protects against atherogenic stimuli. By contrast, low flow, oscillatory flow or other flow patterns that involve changes in direction and magnitude of flow, induce a pro-inflammatory, pro-thrombotic state characterized by high cell turnover (both cell proliferation and cell death) compared to cells in static medium. Thus, flow patterns that mimic those are found at atherosclerosis-prone or resistant regions of arteries, either inhibit or promote events involved in development of atherosclerosis, respectively. 

## 4. Endothelial Cells and Blood Flow

EC regulate, in coordination with vascular smooth muscle cells, the blood flow to tissues, in conjunction with many elements circulating into and out of tissues and with local vasoregulation [59]. This is further facilitated by the responsiveness of EC to vasoactive agents, and by the involvement of the endothelium in the transformation and catabolism of vasoactive agents. They adapt their metabolism according to oxygen tension of the tissues and metabolic needs. By the synthesis and release of endogenous vasodilators such as nitric oxide (NO) and prostacyclin (PGI2) EC play a vital role in the maintenance of cardiovascular homeostasis [60]. Furthermore, the endothelium-derived EDHF as hyperpolarizing factor (e.g., via cytochrome P-450 (CYP) epoxygenase-derived metabolites of arachidonic acid [59,61]) and hydrogen sulfide (H_2_S) have been suggested to be EDRFs as well [62]. 

### 4.1. Regulation by Vasodilation

**Figure 3:**
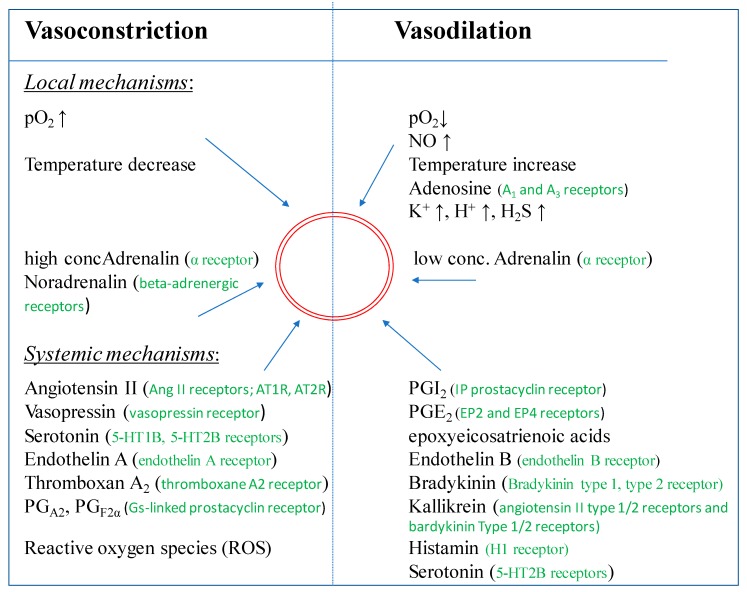
Factors inducing vasodilation and/or vasoconstriction. (The red circle represents a blood vessel).

Endothelial derived relaxing factor (EDRF) was identified as the free radical gas NO seven years after the description of an EDRF by Furchgott & Zawadski [16,63]. NO is not only released upon stimulation e.g., by angiotensin II, acetylcholine, histamine, bradykinin, arachidonic acid, adenine nucleotides, etc., [64] but also plays a significant role in the maintenance of a basal tone (Figure 3). This was demonstrated by the infusion of a NOS inhibitor, like N^G^-Methyl-L-arginine acetate (L-NMMA) leading to an increase of the resting blood pressure [65,66]. NO is not the sole vasodilator which derives from the endothelium [65]. The endothelium also generates PGI_2_, an eicosanoid relaxing the underlying smooth muscle cells through the activation of adenylate cyclase with subsequent generation of cAMP. PGI_2_ is released constitutively by EC [14] and appears to be also involved in the regulation of resting vascular tone. It has been shown to be released in higher amounts in response to ligands binding on the cell surface such as thrombin, arachidonic acid, histamine or serotonin. EC also generate endothelium-derived hyperpolarization factor (EDHF), a hyperpolarizing factor, produced by cytochrome P450 CYP epoxygenases of the CYP2C and CYP2J subfamilies, which is suspected to be an arachidonic acid metabolite. These enzymes metabolize arachidonic acid to various epoxyeicosatrienoic acid (EET) regio- and stereoisomers [65,67]. In multiple in vitro and in vivo studies EETs derived from CYP epoxygenase have been shown to possess other potent biological effects in the renal and cardiovascular systems in addition to EDHF-like properties [68,69]. EETs also inhibit cytokine-induced vascular cell adhesion molecule expression and leukocyte adhesion to the vascular wall [70], inhibit vascular smooth muscle cell migration [71], induce mitogenesis of renal epithelial cells [72] and increase tissue plasminogen activator expression [73]. Recently, it was demonstrated by Wan et al. that the expression and activity of endothelial NO synthase (eNOS) was significantly upregulated by transfection of EC with various CYP epoxygenases [74]. Furthermore, an increased EET biosynthesis was shown. 

Experiments have shown that when NO and Prostacyclin are inhibited vasodilation still occurs [75]. The contribution of EDHF to relaxation seems to be significantly greater in small resistance vessels than in large conduit vessels [76]. Recently, studies could show that EDHF appears to become the predominant endothelium-dependent vasorelaxation pathway, in the absence of the endothelial NOS/NO (as demonstrated in NOS3-knockout mice [77]). An increase in endothelial [Ca^2+^]_I_ activates endothelial derived hyperpolarization (EDH) with subsequent stimulation of two Ca^2+^-sensitive K channels, SK_Ca_ and IK_Ca_ [78,79,80]. These two channels are organized in endothelial microdomains, particularly within projections. These are often directed towards the adjacent smooth muscle, which are rich in IK_Ca_ channels. Other projections are close to inter-endothelial gap junctions. In these gap junctions, SK_Ca_ channels are prevalent. A hyperpolarization of EC occurs by an activation of K_Ca_. An efflux of K^+^ can have a similar effect as diffusible ‘EDHF’ by the stimulation of vascular smooth muscle Na^+-^/K^+^-ATPase and K channels which are inwardly rectifying. Simultaneously, a hyperpolarizing current spreads through myoendothelial gap junctions located on endothelial projections from the endothelium to the smooth muscle. By the spread of ‘conducted’ hyperpolarization along the endothelium of arteries and arterioles to affect conducted vasodilatation the resulting radial EDH is amended. The contribution of EDHF to the endothelium-dependent relaxation is currently actively discussed whether an important feature of a healthy endothelium despite the ongoing debate on its variable nature and mechanisms of action. 

### 4.2. Regulation by Vasoconstriction

On the other hand, endothelium derived factors can also induce vasoconstriction. The vasoconstrictors include angiotensin II (Ang II), thromboxane A_2_, endothelin-1 (ET-1), prostaglandin H_2_ (PGH_2_), and reactive oxygen species (ROS) [44,80,81]. In 1988, endothelin, an endothelium-derived vasoconstricting peptide, was isolated from the cell culture supernatant of porcine EC [82]. The endothelin family is described to consist of three structurally related peptides, ET-1, ET-2, and ET-3 [83]. In the vasculature, the pro-endothelin is released from the basal surface of EC and converted to mature endothelin extracellularly by membrane-bound ‘‘endothelin-converting enzymes (ECE)”. Apparently, endothelin is not stored in EC, but it is synthesized de novo in response to various signals (angiotensin II, cytokines, thrombin, etc.) or physical signals (shear stress, hypoxia, etc.) [83,84,85]. Endothelin is a potent vasoconstrictive agent with long lasting effects.

In inflammation, various functions of the endothelium are pivotal. These functions include increasing permeability, vasodilation, increased leukocyte extravasation, and alterations in the control of coagulation and thrombus formation. While resting, intact endothelium is mainly antithrombotic, however with the activation of the EC by stimuli present in pathological conditions, the endothelium may become pro-thrombotic. Stern et al. demonstrated the presence of receptors for coagulation factors on the intact endothelial surface [86]. In vitro studies identified tumor necrosis factor (TNF) [87], endotoxin [88,89], interleukin-1 [90] and thrombin [91] as agents able to activate EC. Activated endothelium induced tissue factor on the cell surface promoted thrombin formation. 

The exchange of fluids, nutrients and hormones that occurs in the capillaries is enabled by the exposure of a relatively small volume of blood to a large endothelial surface. For this trans-endothelial transport, the active vesicular transport system is important, utilizing plasmalemmal structures (caveolae). On the luminal surface of the endothelium numerous receptors for circulating endogenous substances exist, some of them are located in the caveola, offering the essential means for attachment, endocytosis and subsequent movement across the endothelium into the surrounding tissue. In addition, several macromolecules (e.g., thrombomodulin, Factor VIII antigen) and enzymatic activities (e.g., angiotensin converting enzyme and 5’-nucleotidase) are also localized on the luminal surface of the endothelium. These components on the endothelial surface play important homeostatic roles and regulate the interaction between blood-borne elements and the interstitium.

## 5. Endothelial Cells and Thrombosis

**Figure 4:**
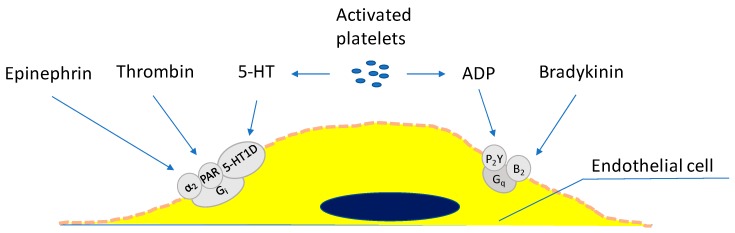
Platelet dependent and independent, receptor-mediated activation of endothelial cells.

In the vascular system of a healthy organism, no harmful interactions between platelets and EC take place, although there is close contact between both cell types [92]. Platelet activation is accompanied by the secretion of mediators which can lead to an activation of EC (see Figure 4), the release of von Willebrand factor, the appearance of P-selectin on the plasma membrane and the production of platelet activating factor and chemokines. Together, these changes initiate a pro-inflammatory and pro-coagulatory situation.

EC secrete various signals and mediators, which are important for the regulation of blood coagulation and platelet functions, in order to prevent platelet adherence and aggregation under normal physiological conditions. Prostacyclin (PGI2) and nitric oxide (NO) are the major antiplatelet agents which are constitutively secreted by EC [93]. Both mediators synergistically increase the cAMP content in platelets, thereby preventing their aggregation [94]. The synthesis of PGI2 and NO is raised as response to a range of agonists (e.g., thrombin or bradykinin) or secreted by aggregating platelets (e.g., ADP, serotonin (5-HT), etc.) and serve to limit the formation of thrombi. In addition to PGI2 and NO, EC also display enzymes at their luminal surface [95]. Ecto-nucleotidase hydrolyze ATP and ADP, both potent platelet aggregating agents, into AMP and adenosine which decrease platelet aggregation [96].

Beyond these effects, EC can maintain blood fluidity. This occurs by promoting the activity of numerous anticoagulant pathways, whereas the protein C/protein S pathway is the most important. By the interaction of thrombin with the EC receptor thrombomodulin the protein C/protein S pathway is initiated, facilitating the activation of protein C [97]. Factors VIIIa and Va, two cofactors which are essential for blood coagulation are inactivated by activated protein C. To be effective, a complex between protein C must protein S (which is synthesized by EC) must be formed [98,99].

The formation of a thrombin and thrombomodulin complex also suppresses thrombin to clot fibrinogen or to activate platelets [100]. A rich source of antithrombin, the main site for inactivation of active thrombin, is provided by heparin-like glycosaminoglycans, which is bound to these glycosaminoglycans. Tissue factor pathway inhibitor is also synthesized by EC [101]. Finally, its elimination from the blood stream occurs by receptor-mediated endocytosis of factor Xa by EC.

Upon vascular injury (damage or detachment of EC), blood components like proteins or cells adhere to the denuded subendothelium—e.g., to collagen fibers or to von Willebrand molecules—become activated and aggregate at the site of injury. This evokes the formation of a stable blood clot preventing excessive blood loss. During the development of thrombosis, the significance of an appropriate control of blood coagulation is obvious. Thrombosis, an occlusive clot within a blood vessel, reduces the blood flow to downstream tissues and organs thereby restricting the delivery of nutrients and oxygen. This can induce necrosis in localized tissue and organ necrosis. Large occlusive thrombi can detach and embolize, then occluding distal vessels. The process of thrombosis followed by embolism is collectively termed thrombo-embolism and can culminate in a variety of local or chronic disorders (e.g., acute arterial thrombosis like in myocardial infarctions or strokes [102]). Similarly, venous thrombo-embolism as major cause of deep vein thrombosis and pulmonary embolism can be triggered by several factors such as disturbed blood flow, hypercoagulable conditions (i.e., pro-coagulant changes in the blood), or EC activation [102].

## 6. Heterogeneity of Endothelial Cells

In general, EC in different regions of the body fulfill similar demands, however some features and response characteristics are distinct. Aird was the first to describe this the heterogeneity of EC [103]. The structural heterogeneity of EC includes variations in cellular morphology (size, thickness and position of the nucleus), as well as in gene expression profile, production of extracellular matrix (i.e., basal lamina components) and finally in cell surface properties. The latter is described by varying compositions of the GCX and the amounts of structural components of the endocytic and transcytosis pathways (i.e., clathrin-coated pits and caveolae, respectively). Furthermore, it also varies in the expression of tight junctions, adherens junctions or gap junctions, the predominant types of intercellular junctions [9,104].

**Figure 5:**
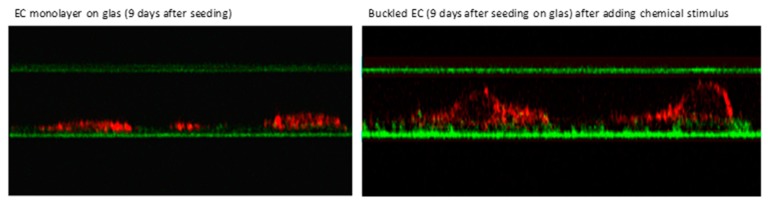
Buckling of human venous endothelial cells in vitro on extracellular matrix pre-secreted by bovine corneal endothelial cells and displayed by confocal laser scanning microscopy (CLSM) induced by addition of an iodinated radiographic contrast medium (Iopromide-370, 30% *v*/*v*) to the cell culture medium [108].

**Figure 6:**
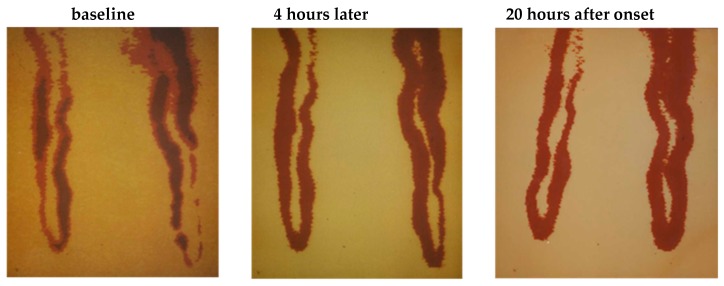
Segmental constriction of a nailfold capillary documented at three time points (final magnification 1:570) [109].

Arteries and veins have different functions. Arteries deliver oxygen and nutrients to tissues, while veins remove cellular waste. Due to these distinctions in function, as well as of local differences in blood pressure, blood flow, pO_2_, and pH, veins and arteries are also different at a cellular and molecular level [104]. Subsequently, EC’s morphologies and functions are adapted to meet these needs in their distinct local environment [105,106,107]. The response of EC to environmental changes or chemical compounds can be very severe. The height or thickness of EC can increase by nearly 100 % (see Figure 5 [108]) sometimes resulting in a blockage of the capillary perfusion (Figure 6, [109]).

Figure 6 shows the complete segmental constrictions of capillary lumina in the venous leg of a single capillary in humans [109]. Approximately 20 h after onset, the luminal diameters have returned to normal resting size.

This heterogeneity of EC promotes to their physiological diversity across the vascular tree [110]. The endothelium reveals regional morphologic differences in intercellular junctions forming the basis of three distinct categories. In most arteries, veins and capillaries of the brain, lungs and skeletal muscle a continuous endothelium, while in certain visceral capillaries, such as the adrenal gland a fenestrated endothelium, and in the sinusoids of the liver, spleen, and bone marrow a discontinuous endothelium is found [111,112]. Continuous endothelium is held together by tight junctions and is anchored to the basal membrane. A continuous sheet of tight junctions-coupled ECs is characteristic of brain, retina, skin, lung and muscle capillaries, while the endothelium covering the inner surface of exocrine and endocrine gland, intestinal villi, kidney glomeruli and subpopulations of renal tubules are porous. These 70 nm wide p is foundores, termed fenestrae, are sealed by a 5 to 6 nm non-membranous diaphragm [9]. Moreover, sinusoidal vascular districts, such as liver, spleen and bone marrow, exhibit larger fenestration (100–200 nm) among ECs, which are devoid of diaphragm and associated to a poorly formed basement membrane [9,80]. In addition, the organization of the gap junctions in the arteries, arterioles, capillaries, and veins appear to be different in these different organs. Morphologic differences also exist in size and thickness of ECs (aortic ECs are 1 µm thick) when compared to those of the capillaries (0.1 µm) and veins (0.1–0.2 µm) [9]. These differences extend to the content of subcellular organelles, for example, Weibel-Palade bodies are abundant in the pulmonary vasculature, as compared to the thoracic aorta (where they are absent) and myocardial capillaries (where they are rare) [113]. Differences also exist in the density of plasmalemmal vesicles, with the highest density in capillaries [9]. These morphologic features between distinct intra- (large versus small vessels) and inter-organ may reflect altered functional characteristics. Especially, EC in brain capillaries that differ from capillary EC in all other organs regarding anatomy and function. Anatomically, the EC of the brain capillaries differ from those in the periphery by a lack of fenestrations, an increased mitochondrial content, minimal pinocytotic activity and the presence of tight junctions; for a detailed description see [114]. In line with this idea, differences in the biochemical composition of EC from various vascular beds were described. One example reported is the distribution of anionic and cationic binding sites on the plasmalemma of EC, which appears to be non-random and may vary in cells in different vascular beds [55].

Consequently, EC in the various vascular beds respond differently to signaling molecules. Comparative studies with freshly isolated EC from coronary micro-vessels, coronary arteries and veins showed regional differences in the capacity of prostaglandin synthesis [56]. Results with short-term cultures, analyzing biochemical and cellular physiological properties in these cells, also suggest some differences [56]. Johnson et al. [58] found that arterial EC had three to five times more angiotensin converting enzyme activity than cultured venous EC. Significant differences are not always obvious, e.g., only little variations have been noted in the protein kinase activities or endogenous substrate content of cultured bovine EC from the pulmonary artery compared to bovine aortic EC [115]. However, differences in morphology and tissue-type plasminogen activator (t-PA) were observed between EC from adult arteries and veins. Firstly, the cells had differing potentials to be propagated as a healthy monolayer. While a homogeneous population of small diameter vena cava cells was retained for 35 population doublings the diameter of aortic EC increased after 8 to 19 population doublings. Secondly, the amount of secreted t-PA varied. EC from the Vena cava produced four times more t-PA than EC from the aorta, and 20-fold more than EC from umbilical artery or vein. Thirdly, the release of Urokinase-type plasminogen activator (u-PA) antigen varied. No u-PA antigen was detectable in the conditioned medium of primary cell cultures of the human aorta and vena cava or of early passage cell cultures from the vena cava. After prolonged sub-culturing, vena cava cells started to secrete u-PA. However, EC from the adult arteries like aorta and others started to secrete u-PA after one to four passages, in parallel to the occurrence of enlarged EC.

In addition, the response of cells from various vascular beds to inflammatory cytokines and vasoactive agents differs, where the postcapillary venules response is most pronounced. Snopko et al. reported that microvascular EC, grown as typical monolayers, producing significantly less prostacyclin than arterial or venous EC after stimulation with serum [116].

Moreover, the response of venous and aortic EC to chemical agents can differ, too [117]. Functionally confluent venous EC on extracellular matrix secreted large amounts of prostacyclin in reaction to iodinated contrast media demonstrating a clear perturbation of the ECs in contrast to arterial EC cultures. The prostacyclin release from arterial ECs exposed to Iodixanol was more than 10-fold higher compared to arterial ECs exposed to Iomeprol. Coinciding with the lower prostacyclin release, decreased cell-cell contacts associated with an increase of denuded areas in confluent cell monolayers were very subtle in arterial ECs after their incubation in culture media supplemented with radiographic contrast media, which was in clear contrast to the marked changes in venous EC [118].

A comparison of cultured EC from arteries, veins, and micro-vessels revealed that not only arteries and veins but in particular, microvascular EC displayed distinct differences showing specific genomic properties and other differences at the tissue level [106]. This is visible in the distinct expression of surface receptors, such as the microvascular-specific α1β1-integrins [119] and plasma-lemma vesicle-associated protein-1, a leukocyte trafficking molecule recognized by the antibody Pal-E [120]. In EC of cerebral micro-vessels, a tissue-specific expression of γ-glutamyl-transpeptidase and monoamine oxidase was seen.

EC in culture may not display the characteristics seen in vivo in the absence of their typical basement membrane [57], although constitutive phenotypic expression may reflect the surface upon which this EC type resides [121,122]. Thus, results from cultured EC models should be regarded with some caution until verified in vivo.

An additional problem with these models was shown to be the difficulty in obtaining and successfully culturing capillary EC from different vascular beds. As of now, this is a limitation for comprehensive comparative studies of the capillary endothelium. There are further limitations when artificial substrata like glass, polymers or metals are used which are foreign to EC. The development of more sophisticated culturing conditions could contribute to the identification of biochemical and functional features, which are unique to the endothelium of specific organs, and may provide insight into interactions between toxins and the vascular bed.

The thickness of the glycocalyx varies tenfold from several hundreds of nanometers to several micrometers throughout the vasculature [23]. Differences in thickness of the glycocalyx were not only described between different vessels (e.g., the thickness of the glycocalyx in the sinus region of the internal carotid artery was lower than in the common carotid artery [34]), but also varied significantly even within a single vessel [23]. In addition, morbidities or therapeutic regimes can change the thickness of the glycocalyx markedly, so that this feature cannot be used to trace the origin of EC in the vascular tree.

## 7. Endothelial Cells and Angiogenesis

**Figure 7:**
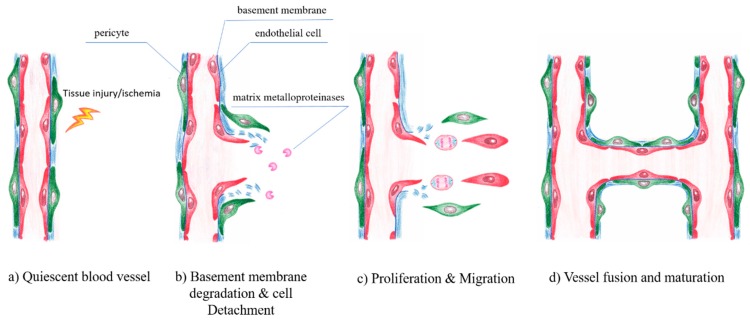
Angiogenesis in brief. VEGF initiates assembly of endothelial cells, PDGF-BB recruits pericytes, whereas angiopoietin-1 and TGF-β stabilize the nascent vessel.

Under normal physiological conditions, EC remain in a quiescent state. However, in response to tissue injury or hypoxic conditions, ECs can more or less rapidly form new vessels by a highly orchestrated process of vessel sprouting, called angiogenesis [123]. Angiogenesis is entirely sustained by local ECs and does not require the mobilization of circulating endothelial progenitor cells [124,125]. Angiogenesis is a complex sequential process of vasodilatation, degradation of basement membrane, EC migration, chemotaxis, increasing vascular permeability, eventually EC proliferation and vessel formation. Several growth and transcription factors control the fine-tuned balance of angiogenesis. The capillary plexus is remodeled by sprouting, microvascular growth and fusion into a mature and functional vascular bed during angiogenesis [126,127]. Angiogenesis also includes vessel penetration into avascular tissue regions and is critically dependent on the essential interactions among EC, pericytes [128], inflammatory cells such as macrophages [129,130] and other adjacent cells associated with the extracellular matrix (ECM) and vascular basement membrane (BM). In contrast to embryonic development, adult angiogenesis is closely related to a number of physiologically relevant processes e.g., wound healing [131,132] or pathophysiological conditions like inflammatory disorders [133], cancer [134] or diabetes mellitus [135,136] (Figure 7).

Angiogenesis is strongly associated with the vascular basement membrane (BM) and the extracellular matrix (ECM). In addition, the interaction between cells and the ECM is required. The first step involves the opening of existing capillaries and partial degradation of surrounding ECM allowing cell infiltration. In initiating this process, EC adopt a proteolytic phenotype and begin to break down the basement membrane by matrix metalloproteases (MMP) [137]. MMP and their endogenous inhibitors, tissue inhibitor of MMPs (TIMPs), regulate the extracellular matrix degradation, remodeling, cellular adhesion and cell growth [138,139,140]. In this process, EC lose their contact with basement membrane laminin and become exposed to interstitial collagens, activating signaling cascades responsible for cytoskeleton reorganization and sprouting morphogenesis [141].

In 1990, Jung et al. could document a case of angiogenesis in a human [142]. The in-vivo occlusion of a cutaneous capillary and the complete time period of the growth of a new capillary could be documented for the first time. A case report described results of a routine examination and the follow up of a young woman who complained of pain typical of Raynaud’s disease. A fresh capillary occlusion was seen in the first check-up. Regular examinations followed in order to investigate the further course of the occluded capillary.

After fifty-three days a growth of a new capillary after capillary occlusion was observed. Over a period of 47 days, the mean capillary growth velocity was measured with 4.7 µm/day. Between the 85th and 98th day after occlusion a maximum growth rate of 10 µm/day was found. A minimum growth rate of 2 µm/day occurred during the first 11 days.

**Figure 9:**
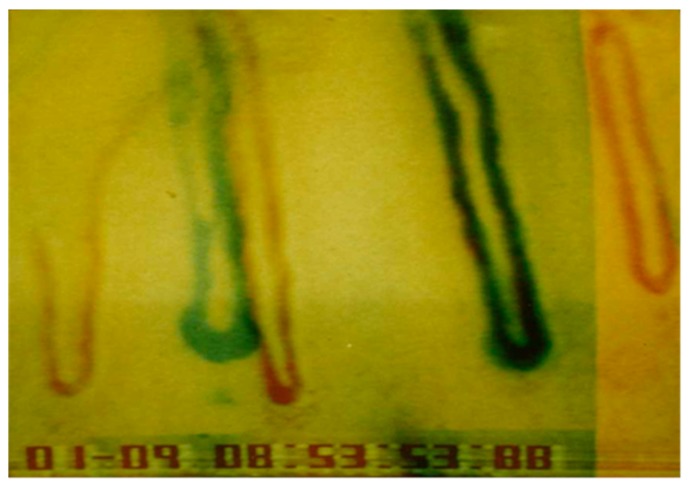
Overlay of the former and the remodeled capillary beds by means of a Helmert transformation. (The picture in red shows the capillary bed before occlusion, superimposed by the picture in green with the newly grown capillary (Reprinted with the permission of [127], S. Karger AG)).

An important question to consider was whether the old capillary channel was used again or a completely new channel was formed. Differences can be seen by superimposition of the picture after growth of the new capillary (22.4.1990, Figure 8) and the picture before capillary occlusion (9.1.1990, Figure 7). Both pictures were processed and superimposed so, that the neighboring capillaries, which had remained unchanged during the whole observation time, were used as a reference frame. This was accomplished mathematically by the Helmert transformation of Cartesian coordinates. Figure 9 shows a picture of the nailfold capillaries before capillary occlusion superimposed by a second picture of the identical capillaries after capillary occlusion with the newly grown capillary. The superimposed capillary bed is dark (result of red and green overlay). On the left side is the former capillary bed (red loops) and close to it the remodeled capillary bed (green loops). The picture suggests that the former capillary bed was not used again, but that a new one formed.

The capillary was always found to be well perfused during growth. In relation to the neighboring capillaries, the permeability of the newly grown capillary appeared to be normal (tested by sodium fluorescein permeability [143]). The pericapillary halo of the three capillaries within the visual field was sharply limited and nearly of the same size. Thus, the physiological functionality of the new capillary with regard to permeability and perfusion was shown.

Numerous genetic and molecular signals were identified over the last decades to regulate this process [144] and many factors impact the generation of new blood vessels, including pro-angiogenic and also angiostatic factors. Angiogenesis was shown to rely on a migratory “tip”-cell which guides the vessel sprout at the forefront, and trailes proliferative “stalk”-cells that elongate the sprout [145]. The initiation of angiogenesis is mainly driven by VEGF, the key angiogenic vascular growth factor, which induces the specification of ECs into tip- or stalk-cells, via binding to the VEGFR-2 [123]. The EC most sensitive to VEGF, co-determined by its higher VEGFR-2 expression relative to that of its neighboring cells [146], front becomes a tip-cell at a newly forming vascular, which guides the vessel sprout at the forefront, forming numerous filopodia that scan the environment for angiogenic cues [118]. This specific capability of the tip- cells occurs via a notch-mediated lateral inhibition mechanism [144,147]. The VEGF/VEGFR-2 activated ECs express the Notch-ligand DLL4 [148]. In order to prevent excess angiogenesis DLL4 functions as a dampening mechanism promoting orderly development of newly growing vessels [123,149]. Neighboring ECs are promoted to become proliferating stalk- cells by the lower responsiveness to VEGF [150]. A lumenized vessel that perfuses the previously avascular tissue is formed, as soon as two tip cells of opposing branches anastomose. Thereafter ECs adapt to a non-migratory and quiescent phenotype, organizing themselves in a continuous monolayer allowing the perfusion with blood [151]. The last step in this cascade is the pruning of excess endothelial tubes, the stabilization of the remaining tubes by pericytes and their maturation into capillaries.

A common attempt to repair or replace biological tissues is the implantation of biomedical devices or synthetic matrices. Often, an adverse foreign body reaction occurs in the vicinity of implanted devices [152], associated with inflammatory reactions which can induce vessel growth around the implants [153]. This is a result from persistent inflammatory stimuli, which mediate series of cellular alterations by various surrounding cells. In such an environment, lymphocytes, macrophages, mast cells, and their granular products together with degradation products of the implant contribute to the formation of foreign body giant cells and the development of a dense layer of fibrotic connective tissue, the so-called capsule [154,155]. These reactions can vary in intensity and severity, depending on the chemical and physical nature of the implanted materials [156]. Very recently, Ullm et al. could show that e.g., gelatin-based hydrogels induced a very low foreign body response with a low inflammatory response, comparable to the response in sham-operated animals. Angiogenetic processes and vessel outgrowth occurred in the surrounding capsule [157], supplying the invasion of cells and removing cellular debris and degradation products.

## 8. Hematogenous Metastasis

Metastasis distant from the primary tumor are created through tumor cell infiltration into the blood circulation and transport to other regions of the organism via the blood flow [158]. Hematogenous metastasis typically occurs in tissues/organs localized downstream. For tumor cells to emigrate out of the vasculature and settle, they can:

(i) express specific membrane receptors (e.g., adhesion molecules, sialic acid ligands, etc.), whose ligands would be found most favorably on EC of the target organ, or

(ii) a lump of tumor cells can locally induce blood coagulation, creating an embolus mixed of blood and tumor cells which favors attachment of the embolus to EC (unspecific gatekeeper effect for the emigration of tumor cells from the blood circulation [159]).

The organ-specific heterogeneity of EC favors settling of tumor cells and metastatic spread in specific organs.

## 9. Conclusions

This review focuses on the morphology and function of endothelial cells, particularly their differences in different localizations around the body paying attention specifically to their different responses to physical, biochemical and environmental stimuli considering the different origins of the EC. It has to be kept in mind, that the results presented here were gathered either from in vitro analyses or from animal studies. Ethical considerations do not allow to study endothelial cells from different vascular regions in humans. This is true for healthy persons and especially for patients with different kinds of diseases. That is why, the transfer of these results to the situation in humans has to be regarded with caution. In general, it is of utmost importance for the conduct of in vitro studies to generate culture conditions, e.g., cell culture media, cell substrates and cell environments, which create growing similarity to the situation in situ/in vivo.

A further deepening of the understanding of the endothelial cell biology is essential for the elucidation of specific mechanisms in tissue engineering and regenerative medicine or for novel approaches in the treatment of cardiovascular diseases.

## Figures and Tables

**Figure 8:**
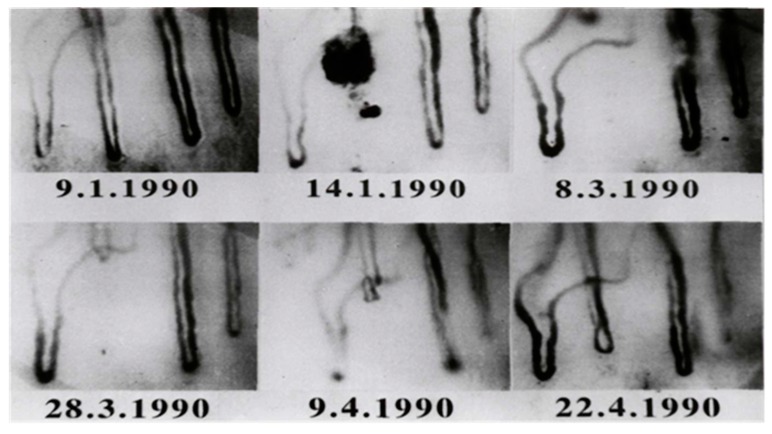
Capillary projection before occlusion and at different phases of capillary remodeling (Reprinted with the permission of [142], S. Karger AG).
